# Experimental Characterization of NURSE, a Device for Arm Motion Guidance

**DOI:** 10.1155/2018/9303282

**Published:** 2018-07-03

**Authors:** Betsy Dayana Marcela Chaparro-Rico, Daniele Cafolla, Marco Ceccarelli, Eduardo Castillo-Castaneda

**Affiliations:** ^1^Instituto Politécnico Nacional-CICATA Querétaro, Cerro Blanco 141, Colinas del Cimatario, 76090 Santiago de Querétaro, QRO, Mexico; ^2^Laboratory of Robotics and Mechatronics (LARM), University of Cassino and Southern Lazio, Via Di Biasio 43, 03043 Cassino, Italy

## Abstract

This paper presents an experimental characterization of NURSE, a device for arm motion guidance. The laboratory setup and testing modes are presented to explain the experimental procedure. Two exercises for the upper limb exercise are used to test the NURSE behaviour, and successful results are presented. Trajectories and linear accelerations are tested when the device performs the two exercises without and with load. In addition, torque and power consumption are considered to check the NURSE behaviour.

## 1. Introduction

Every year, 15 million people worldwide suffer a stroke; more than 70 percent must deal with mobility impairment and cognitive disabilities [1]. Additionally, the arm mobility can be affected by neurological, muscle, and joint diseases [2]. Lymphatic and vascular disorders can also reduce arm mobility [3]. On the contrary, the arm mobility can be also affected by traumatic and overuse injuries of the shoulder, elbow, and wrist [3, 4]. In such a case, exercises are necessary to recover a suitable range of motion by strengthening, flexing, and extending the muscles and the joints [5]. However, the number of trained human therapists who can provide this support is limited, while the demand is growing, particularly in elderly people [6, 7]. The required exercises for an assistive therapy should be designed by a specialist according to the medical diagnosis, and it can be vary from a specialist to another [3, 8, 9]. However, all exercises start from the basic movements of the human arm seen in [3, 4, 8, 10, 11]. During a traditional exercise, the specialist assists the limb motion. However, it is difficult for the therapist to keep the same quality of motions during long-therapy sessions. In addition, the motion cannot be controlled, and a feedback of the patient evolution is difficult to obtain. While there remain a number of tasks that only human therapists can perform, many rehabilitation exercises are mainly highly repetitive. This is where robotic systems are useful since they can reproduce the same task countless times, with precision and accuracy without fatigue or loss of attention [12]. It has been proved that use of robotic systems benefits the rehabilitation process [13, 14]. In addition, the use of robotic systems reduces the recovery time by 30% [13]. Several devices have been developed for arm motion assistance. However, there are several issues to solve in the existing robotic devices such as they are costly and they have bulky structures very difficult to adjust to the patient arm.

The existing devices for arm motion assistance can be classified into three groups: nonactuator devices, exoskeletons, and end-effector devices. The nonactuator devices are frequently used by rehabilitation centers since they have significant lower costs, are easier to use, and are inherently safe. An example is the handboard to trace the number 8 [15], and the mechanism is composed of a roller skate for the arm support, a table with a guide with an 8 shape, and pieces of different weights to apply resistance to the motion during the therapy. However, the mechanism offers only one exercise, and the arm motion is not controlled. Another example is the skateboard [16, 17]. The skateboard is a known mechanism composed of a board with wheels that allow movements on a horizontal plane. The patient should perform the movements by himself/herself. Since the skateboard is a cheap mechanism, it is widely used. However, by using the skateboard, the therapy motion cannot be controlled. However, the nonactuator devices do not have movement correction. Within the exoskeletons group, ArmeoPower can be found [18]. ArmeoPower has six degrees of freedom to perform 3D motions and a graphical interface for virtual interaction. However, ArmeoPower is difficult to wear, costly, and has a bulky frame. Another exoskeleton is MEDARM [19], which can assist the arm motion on a horizontal plane; it is actuated by cables and has 3 degrees of freedom. The MEDARM exoskeleton is adjustable for users of different sizes. However, MEDARM needs a bulky frame structure, is difficult to transport and construct, and has been proposed only to assist the right arm, and it is difficult to align the exoskeleton joints with the human arm joints. Another exoskeleton named “CAREX” is proposed in [20]. The exoskeleton is actuated by seven cables and has five degrees of freedom. However, CAREX needs a very huge structure to support the seven motors that move the cables. In addition, the cables can be dangerous for the subject since they move close to his/her head. Other exoskeletons with similar disadvantages to ArmeoPower, MEDARM, and CAREX can be seen in [21–23]: in [21], an exoskeleton is proposed to assist just the shoulder motion, but it is difficult to wear; in [22], an exoskeleton is proposed to assist the elbow and wrist joints, but it has a bulky frame structure and it is not comfortable to use since the frame must be placed in the middle of the patient's legs so that the arm gets a the proper position; in [23], an exoskeleton named “ARMin III” is proposed, but like ArmeoPower, it is difficult to wear and has a bulky frame. As seen in [18–23], the main issues in the exoskeletons are that the expostulations have joint axes fully determined as well as physiological movements, but robot axes have to be aligned with anatomical axes and are very difficult to transport, construct, and wear. In addition, the exoskeletons are very difficult to adapt to different anthropometric sizes. An example of an end-effector device can be seen in [16]. The device is based on a planar parallel mechanism 3RRR. The device can assist the arm motion on a horizontal, vertical, or inclined plane by performing several trajectories within its workspace. However, the device has large links and presents stiffness problems. In [24], an end-effector device is proposed to assist the arm motion. The forearm of the user is supported by an end-effector device, and the device can assist the shoulder/elbow flexion and extension without other trajectories. The disadvantage of this end-effector device is that it covers a small workspace and offers few types of exercises. In [25], a portable end-effector device is proposed for arm exercises on an inclined plane. The device is composed of two actuators that are actuated by cables and a hand grip device. The device trajectories are limited by four guides that constrain the end-effector movement along straight lines, and the device cannot perform other types of exercises. In [26], MIT-MANUS is presented, a commercial and known device for arm therapy that has been developed in the early 1990s. MIT-MANUS is principally composed of a five-bar mechanism and a modular end-effector. The robotic arm helps in the shoulder and elbow motion on a horizontal plane, and the modular end-effector allows the movements of the wrist joint. Currently, MIT-MANUS has a clinical version that is named “InMotion ARM™” as pointed out in [27]. However, the device has a reduced workspace in terms of the range of possible motions. Furthermore, the device is not portable and it requires to be operated by highly trained personnel. Another end-effector device named “REAplan” is presented in [28]. The device is based on the Cartesian mechanism with a handle that is moved on a horizontal plane to assist the arm motion. However, REAplan has a bulky and heavy structure so that is difficult to transport. In addition, it has a reduced workspace in relation to the required link sizes. However, the end-effector devices present advantages with respect to the exoskeletons such as they present a simple structure and control and they are easy to adjust to the patient.

As seen in the above examples, the main issues to consider about the existing devices for arm motion are that the devices with a large workspace are very difficult to transport, construct, and wear as seen in [18–23]; the existing portable devices cover a small workspace [16, 24–28] and offer few types of exercises [24, 25]; and the widely used basic mechanisms do not have motion control during the therapy or they perform a single trajectory as seen in [15, 17].

In order to solve the above issues, NURSE (cassiNo-qUeretaro uppeR-limb aSsistive dEvice) was developed as an alternative solution for arm motion assistance with advantages over the existing devices. NURSE is an end-effector device composed of a mechanism, a controller, and a user interface. NURSE is based on a mechanism of 2 degrees of freedom whose workspace is amplified by using a pantograph. NURSE can assist the arm motion during a rehabilitation therapy and the arm motion of elderly people during an exercise. The main advantages of NURSE are presented in this paper together with the experimental characterization.

## 2. Exercises for Arm Motion Guidance

**Figure 1:**
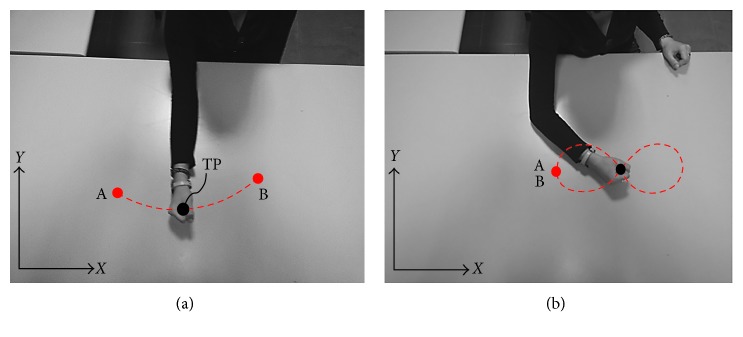
The two considered exercises for upper limb rehabilitation and exercise: (a) exercise no. 1 to treat the shoulder joint; (b) exercise no. 2 to treat both shoulder and elbow joints.

In order to assist the arm motion during a therapy, two exercises for upper limb rehabilitation and exercise have been designed by the authors as reported in [29] (Figure 1). The considered exercises can be used in patients recovering from injuries and neurological, muscular, and joint diseases. Moreover, they can also be used for the arm exercise by elderly people.  Figure 1(a) shows exercise no. 1 that has been designed to treat the shoulder. The exercise consists of performing a horizontal shoulder flexion by tracing the trajectory in red dotted lines with a tracing point (TP) from the point A to the point B (Figure 1(a)).  Figure 1(b) shows exercise no. 2 that has been designed to treat both the shoulder and elbow joints. The exercise consists of tracing the number *8* with the TP. Exercise no. 2 starts and ends in the same point. Since the path to trace the number *8* is complex, it is also used as a reference trajectory to evaluate the behaviour of robots that perform human tasks [30].

**Figure 2:**
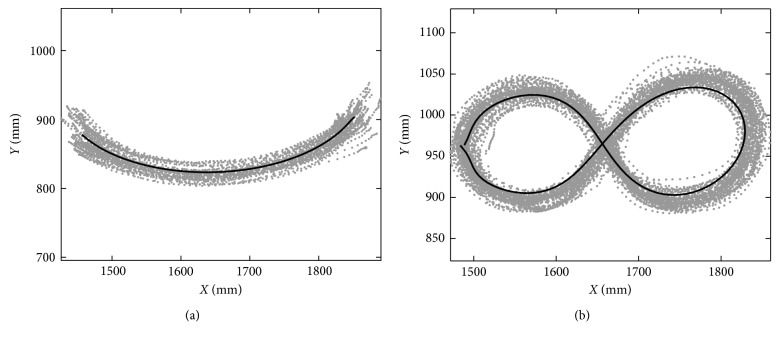
Reference trajectories generated by regression analysis (in black) and the trajectories acquired from the 12 subjects: (a) trajectories for exercise no. 1 to treat the shoulder joint; (b) trajectories for exercise no. 2 to treat both the shoulder and elbow joints.

The procedure for the motion design of the considered exercises is explained in [29]. The reached coordinates of the TP with respect to an *XY* reference frame were used for the design motion. Since in an assistive therapy, the patient's hand is guided by a specialist to perform a desired exercise, it is assumed that a device for motion assistance should perform the path of the same exercise. A Kinect vision system [31] was used to carry out the data collection of the arm motion from 12 subjects that performed the above exercises. The subjects performed each exercise during 12 repetitions. From the collected trajectories, a reference trajectory was generated for each exercise by using regression analysis as reported in [29].  Figure 2 shows the trajectories generated for each exercise and the trajectories acquired by the Kinect vision system. It is important to notice that other arm exercises have also been designed in [29].

## 3. Laboratory Setup and Testing Modes

**Figure 3:**
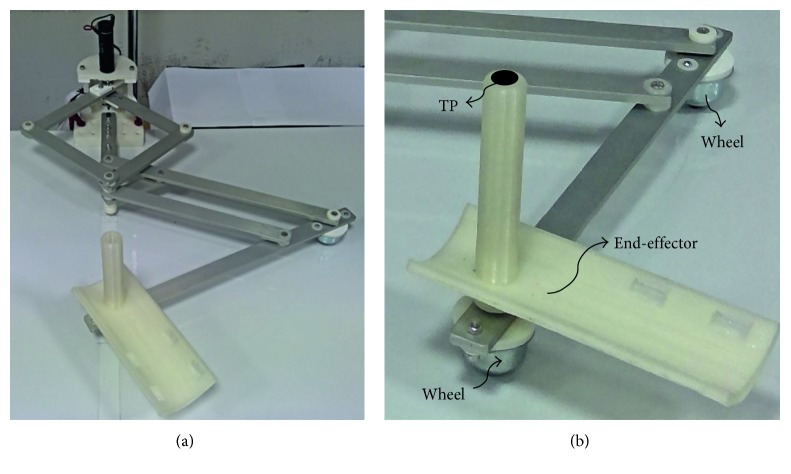
NURSE: (a) a prototype; (b) the tracing point (TP) on the end-effector and wheels.

NURSE has been conceived and designed to solve all the issues that have been mentioned in Introduction, giving the possibility to perform exercises useful for physical therapy or rehabilitation, for treatments of injuries or diseases, for prevention of injuries or diseases, or for physical exercising [32] (Figure 3(a)).

The proposed device is composed of a linkage structure that is driven in planar movements by two actuators. Two wheels are used to support the NURSE structure (Figure 3(b)). The used wheels have omnidirectional balls of stainless steel, and they can support a load of 25 kg each. In addition, an end-effector has been designed for a comfortable grasping of the user. Figure 3(b) shows the tracing point TP on the NURSE end-effector. The linkage structure is composed of aluminum bars that have a thickness of 6 mm and a width of 25 mm. The mechanism structure weighs 2.6 kg, and it fits into a box of 35 × 45 × 30 cm. More details of the mechanical design of NURSE are explained in [32, 33]. The mechanism can guide both right and left human arms on a plane within a large workspace to follow whatever desired trajectory [32, 33]. The planar linkage structure is characterized by light links for compact design, low-power consumption, and easy portability. The movements that can be performed by NURSE involve the shoulder and elbow of a human arm in an independent way or in a coordinated motion. Since NURSE can perform several trajectories of different sizes, it can be used by people of any age, anthropomorphic sizes, and anthropometric sizes, including children and elderly people as pointed out in [32, 33].

**Figure 4:**
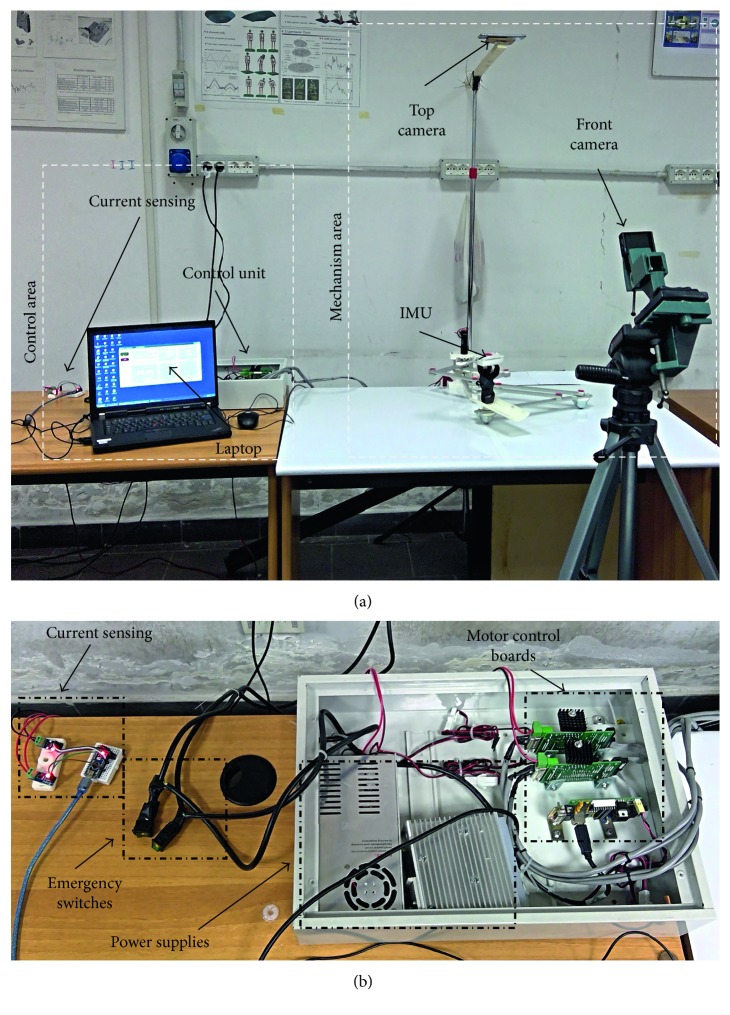
Experiment layout: (a) overview of the lab setup; (b) control area details.

To test the performances and the behaviour of NURSE, some experiments have been carried out at LARM laboratory in Cassino. A specific layout has been designed to allow a satisfactory acquisition of the needed data (Figure 4). In Figure 4(a), it is possible to notice that the area can be divided in two subareas, namely, the mechanism area and the control area.

The mechanism area includes the NURSE together with two cameras. One camera has been installed on the top of NURSE being planar to its workspace, while the other camera has been installed in front of NURSE. Furthermore, an IMU (inertial measurement unit) sensor has been placed on the TP.

The control area consists of a laptop in which an interface sends the positions for the NURSE motors according to a selected arm exercise, the control unit, and the current-sensing modules as in Figure 4(b). Each actuator is connected to a control board that will generate the trajectories to reproduce the selected exercise from the interface. The current-sensing module is composed of two current sensors, and each sensor is connected to each motor. Finally, one emergency switch turns off the motor amplifier, while the second turns off the entire system.

**Figure 5:**
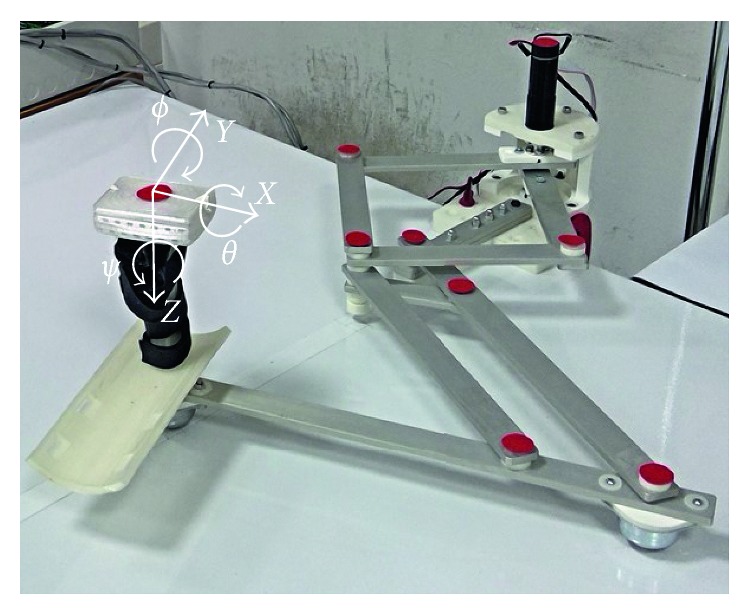
Markers for image processing.

The top camera has been used to track the movement of the TP to validate if the programmed trajectory is satisfactorily reproduced by NURSE; to do so, some markers (red color circles) have been placed on the structure for the motion tracking by image processing (Figure 5). The front camera allows for an overview of the working area.

The placed IMU sensor on the TP can be used to measure the angular displacement in terms of roll (*θ*), pitch (Φ), and yaw (*ψ*) and to acquire the linear acceleration along *X*, *Y*, and *Z-*axes as shown in Figure 5.

The two current sensors based on the Hall effect are used to compute the power consumption and check the behaviour of each actuator.

**Figure 6:**
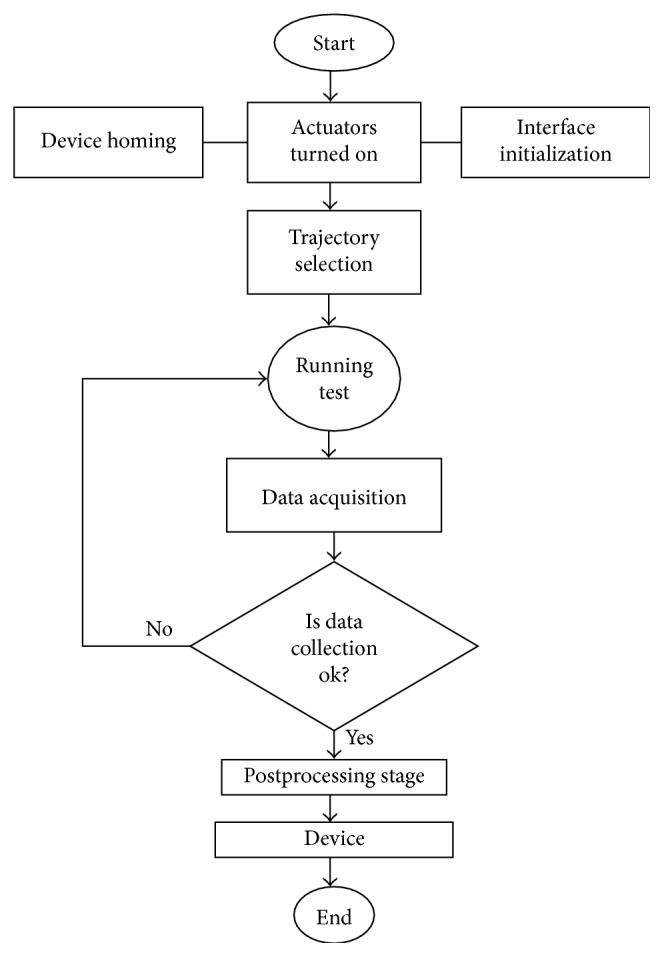
Experiment flow chart.

The experiments are carried out following the flow chart shown in Figure 6. Before running a test, the device is set manually in the home position, the actuator is turned on, and the interface is initialized. After that, the exercise to be performed is selected, the exercise trajectory is sent to the motor control board, and the test runs reproducing the desired task. While the experiment is running, the data are acquired from the cameras and the sensors. When the exercise ends, the data are collected and checked to evaluate if there is any data discrepancy due to sensors or video acquisition failure. In such a case, the test is repeated; otherwise, the postprocessing stage starts and the device characterization is carried out to evaluate the performance of NURSE.

Table 1 shows parameters of the tests that have been carried out in order to characterize the NURSE behaviour. The references trajectories of exercise nos. 1 and 2 in Figure 2 are used to carry out the tests. In test no. 1, NURSE performs exercise no. 1 during three repetitions. In test no. 2, NURSE performs exercise no. 2 during three repetitions. Both tests are carried out without load and with a load of 520 g by using a velocity of 396 °/s. The used load of 520 g is equivalent to 30% of the average weight of the forearm together with the hand, and it has been considered enough for lab experiments. In both tests, the positions of the TP are programmed in the control (*X*, *Y*) as inputs.

After the acquisition, the positions of the TP (*X*_m_, *Y*_m_) are obtained by image processing. The positions of the TP are used to validate if the device is able to perform the programmed trajectory. The linear accelerations of the TP (*a*_*x*_, *a*_*y*_) are acquired by the IMU sensor, and they can be used to evaluate the smoothness of the motion as an important aspect for user safety. Using the acquired motor's current, it is possible to compute the torque of each motor (*τ*_1_, *τ*_2_) and the power consumption (*p*) of NURSE to evaluate if the actuators struggle while replicating the task.

## 4. Test Results

**Figure 7:**
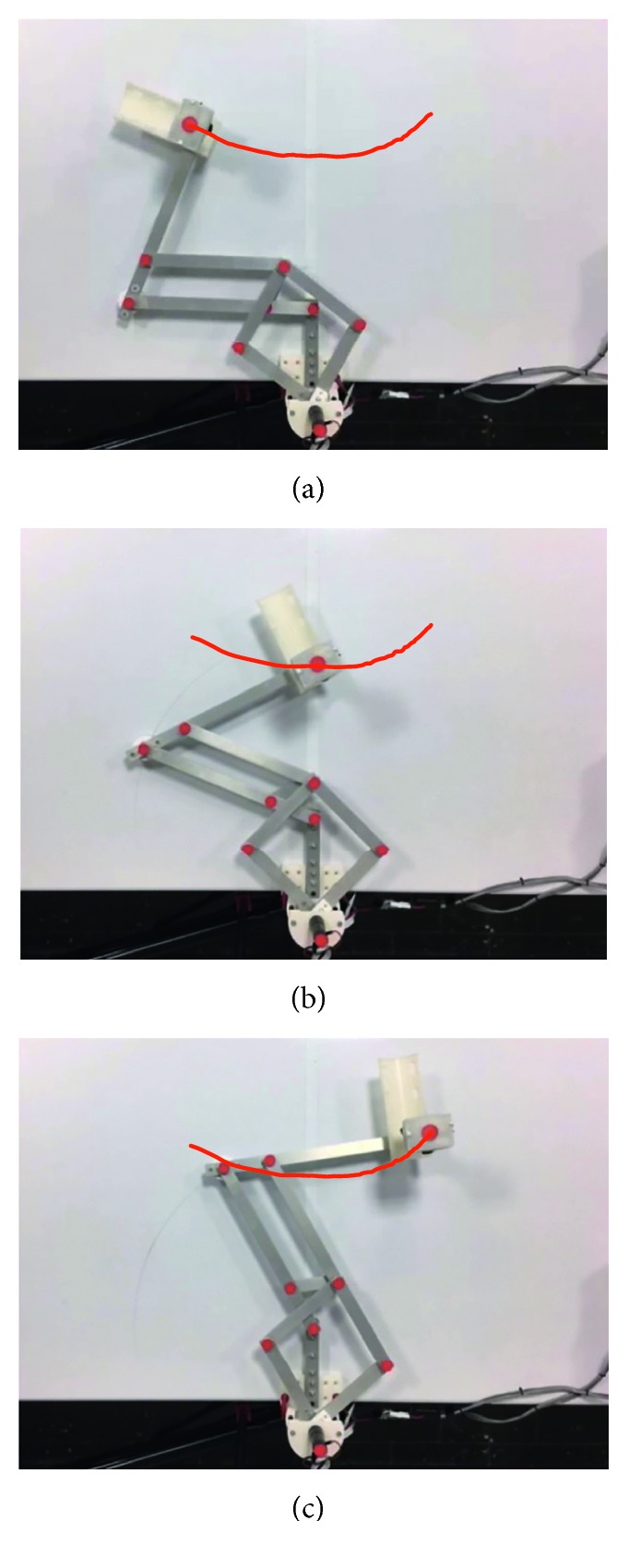
Some snapshots of test no. 1 without load during repetition no. 1 together with the trajectory obtained by image processing (in red): (a) the first sample position; (b) the second sample position; (c) the third sample position.

**Figure 8:**
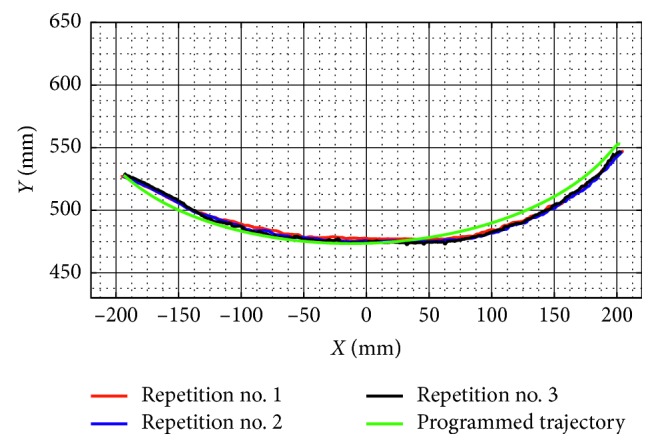
Comparison between the programmed trajectory and the TP trajectories from three repetitions during test no. 1 without load.

Test no. 1 has been carried without load during three repetitions.  Figure 7 shows three snapshots of the video while the test is carried out during repetition no. 1. In addition, Figure 7 shows the trajectory obtained from the marker on the TP.  Figure 8 shows the trajectory programmed in the device and the trajectories obtained from the marker on the TP during repetition nos. 1, 2, and 3. As shown in Figure 8, the trajectories obtained from the marker on the TP are close to the programmed one with a maximum deviation of 10 mm. This deviation is related with the accuracy of the home position since it is set manually. However, the repeatability deviation between the trajectories performed by NURSE has a maximum value of 3 mm for test no. 1 without load.

**Figure 9:**
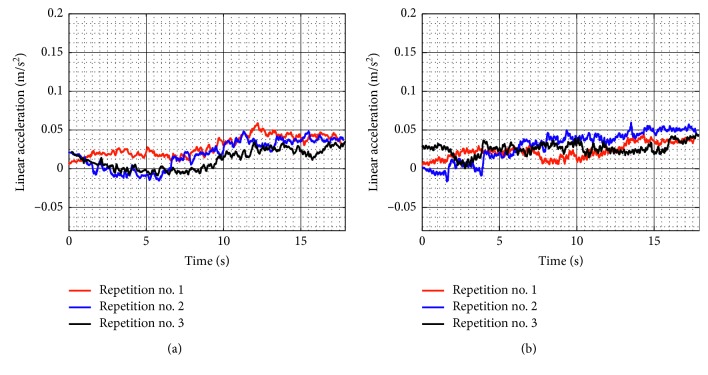
Acquired linear acceleration during test no. 1 without load for the three repetitions seen in Figure 8: (a) *X* linear acceleration; (b) *Y* linear acceleration.

Figure 9 shows the linear accelerations acquired from the TP during test no. 1 when the device is unloaded for the three repetitions as seen in Figure 8. The linear accelerations in *X* have a maximum value of 0.058 m/s^2^ and a minimum value of −0.015 m/s^2^, and linear accelerations in *Y* have a maximum value of 0.059 m/s^2^ and a minimum value of −0.015 m/s^2^. The linear acceleration values in *X* and *Y* are negligible, and it shows that the movement of the TP is smooth. The spikes in the linear acceleration are due to the backlash of the wheels.

**Figure 10:**
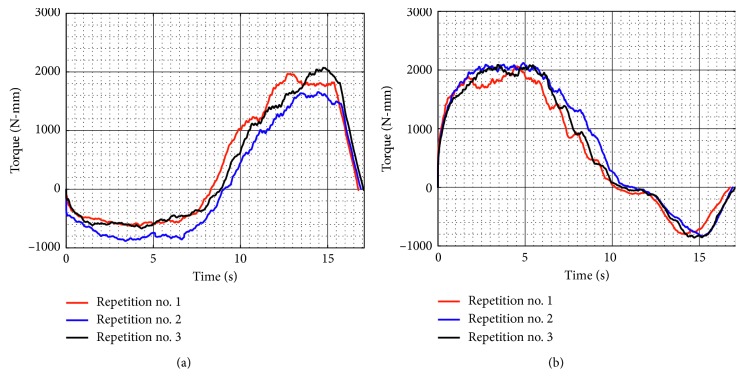
Computed torques during test no. 1 without load for the three repetitions seen in Figure 8: (a) Motor 1; (b) Motor 2.

Figure 10 shows the acquired motor torques during test no. 1 when the device is unloaded for the three repetitions as seen in Figure 8. Motor 1 reaches a maximum magnitude of 2,071 N-mm, and Motor 2 reaches a maximum magnitude of 2,119 N-mm. The torque values confirm that commercial servomotors can be used for NURSE motion. In addition, the torques curves show a symmetrical behaviour between Motor 1 and Motor 2.

**Figure 11:**
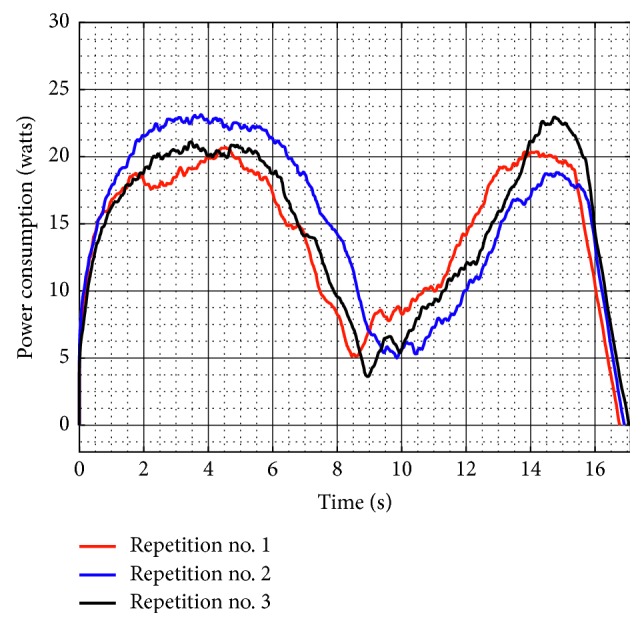
Computed power consumption of test no. 1 without load for the three repetitions seen in Figure 8.

Figure 11 shows the power consumption of NURSE without load during test no. 1. The power consumption reaches a maximum value of 23.130 W. The power consumption values confirm that NURSE works with low-power consumption when it is unloaded.

**Figure 12:**
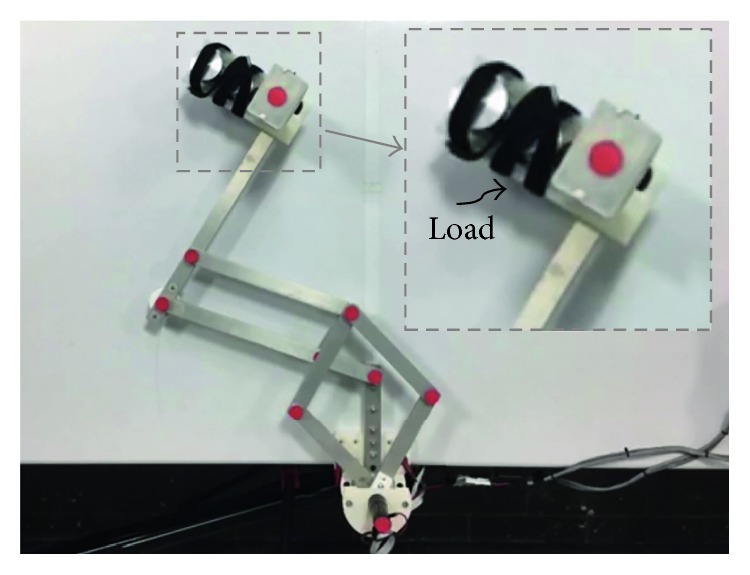
A zoomed view of a NURSE end-effector with a load of 520 g.

**Figure 13:**
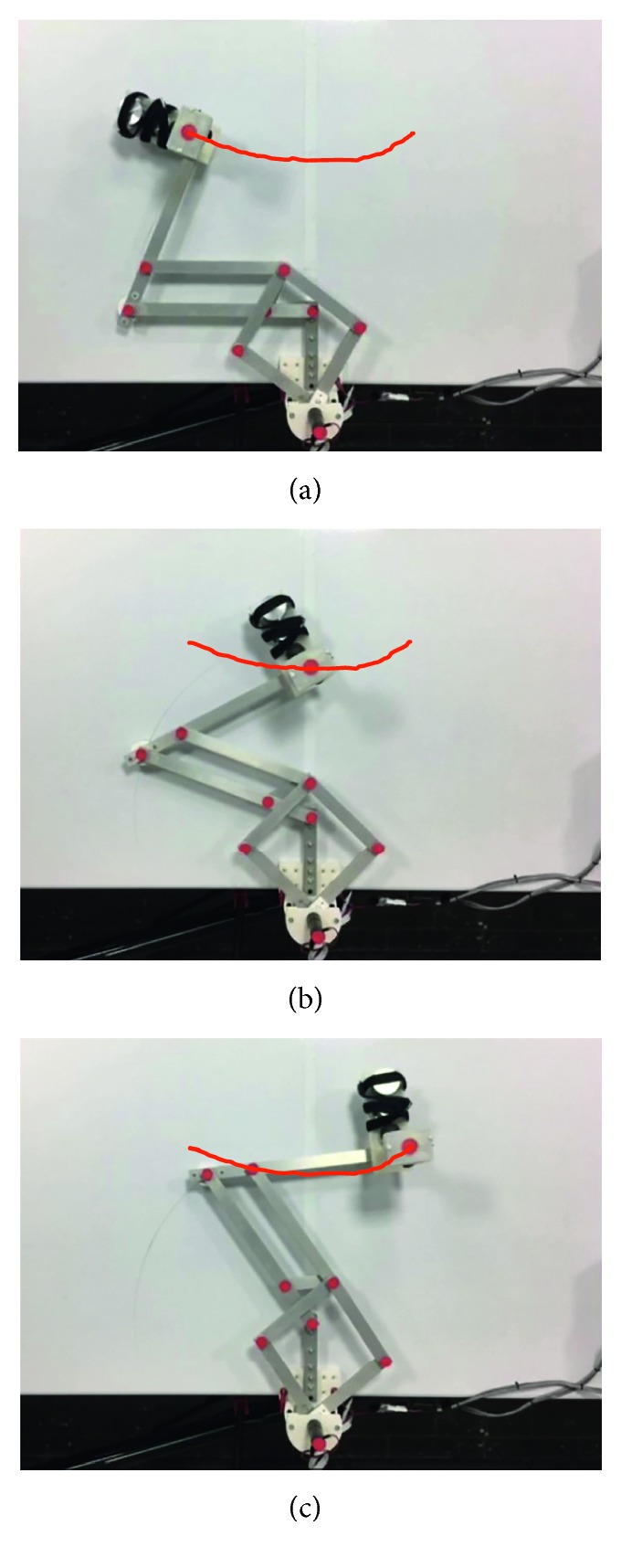
Some snapshots of test no. 1 with a load of 520 g during repetition no. 1 together with the trajectory obtained by image processing (in red): (a) the first sample position; (b) the second sample position; (c) the third sample position.

**Figure 14:**
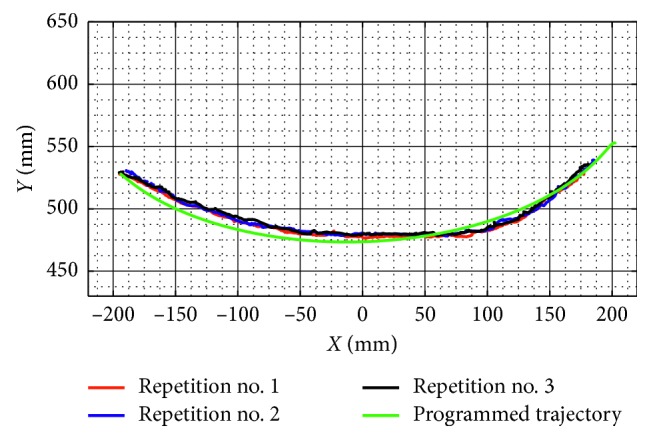
Comparison between the programmed trajectory and the TP trajectories from three repetitions during test no. 1 with a load of 520 g.

Similarly, test no. 1 has been carried out with a load of 520 g during three repetitions.  Figure 12 shows a zoomed view of a NURSE end-effector with the load of 520 g. Some snapshots of the test with the acquired trajectory from the TP during repetition no. 1 are shown in Figure 13.  Figure 14 shows the trajectories acquired from the TP during the three repetitions and the programmed one. When NURSE is loaded in test no. 1, the deviation between the trajectories acquired from the TP and the programmed one has a maximum value of 13 mm. The deviation when NURSE is loaded is 3 mm greater than the deviation when NURSE is unloaded. However, this difference is negligible, and it can also be related with the accuracy of the home position as mentioned above. It is important to notice that the repeatability deviation between the trajectories performed by NURSE has a maximum value of 4.5 mm for test no. 1 with load.

**Figure 15:**
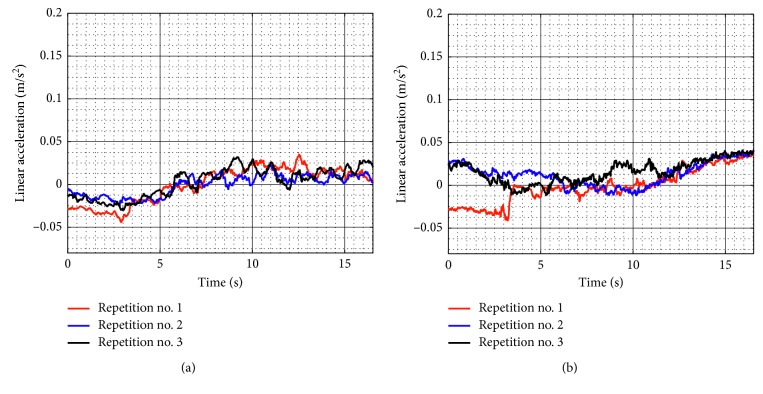
Acquired linear acceleration during test no. 1 with a load of 520 g for the three repetitions seen in Figure 14: (a) *X* linear acceleration; (b) *Y* linear acceleration.

The linear accelerations acquired from the TP during test no. 1 with a load of 520 g are shown in Figure 15. The linear accelerations in *X* have a maximum value of 0.036 m/s^2^ and a minimum value of −0.045 m/s^2^, and linear accelerations in Y have a maximum value of 0.041 m/s^2^ and a minimum value of −0.039 m/s^2^. Therefore, the linear acceleration values in *X* and *Y* are also negligible when the device is loaded. Thus, NURSE can reproduce the exercise of test no. 1 when it is loaded as smoothly as when it is unloaded.

**Figure 16:**
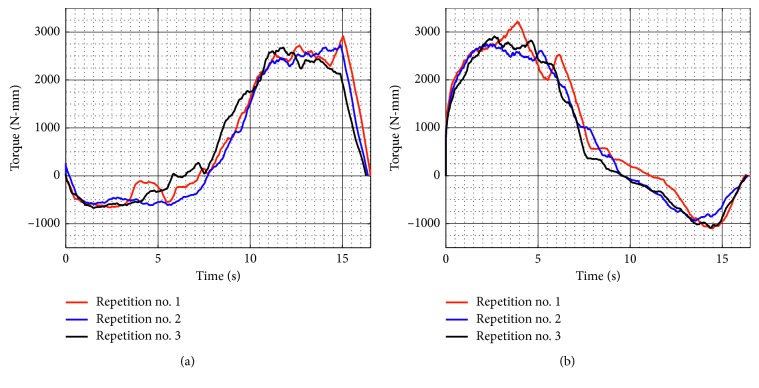
Computed torques during test no. 1 with a load of 520 g for the three repetitions seen in Figure 14: (a) Motor 1; (b) Motor 2.

When the device is loaded during test no. 1, the torque of the Motor 1 reaches a maximum magnitude of 2,926 N-mm and Motor 2 has a maximum magnitude of 3,217 N-mm (Figure 16). As seen in Figure 16, the torque increases around 1,098 N-mm when the device is loaded with respect to the torque when it is unloaded as seen in Figure 10. However, the torque values confirm that NURSE can also be moved by commercial motors in the loaded condition.

**Figure 17:**
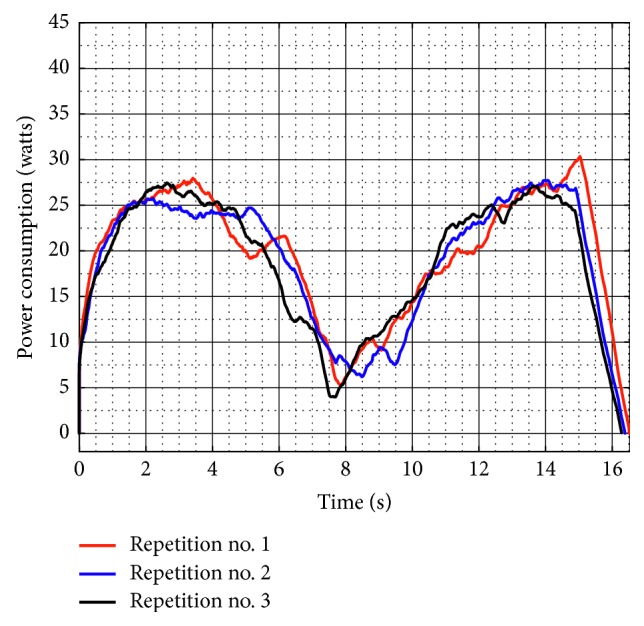
Computed power consumption during test no. 1 with a load of 520 g for the three repetitions seen in Figure 14.

The power consumption when NURSE is loaded has a maximum value of 30.35 W for test no. 1 (Figure 17). Thus, it increased 7.220 W with respect to the power consumption when NURSE is unloaded. Therefore, the NURSE low-power consumption characteristic remains.

**Figure 18:**
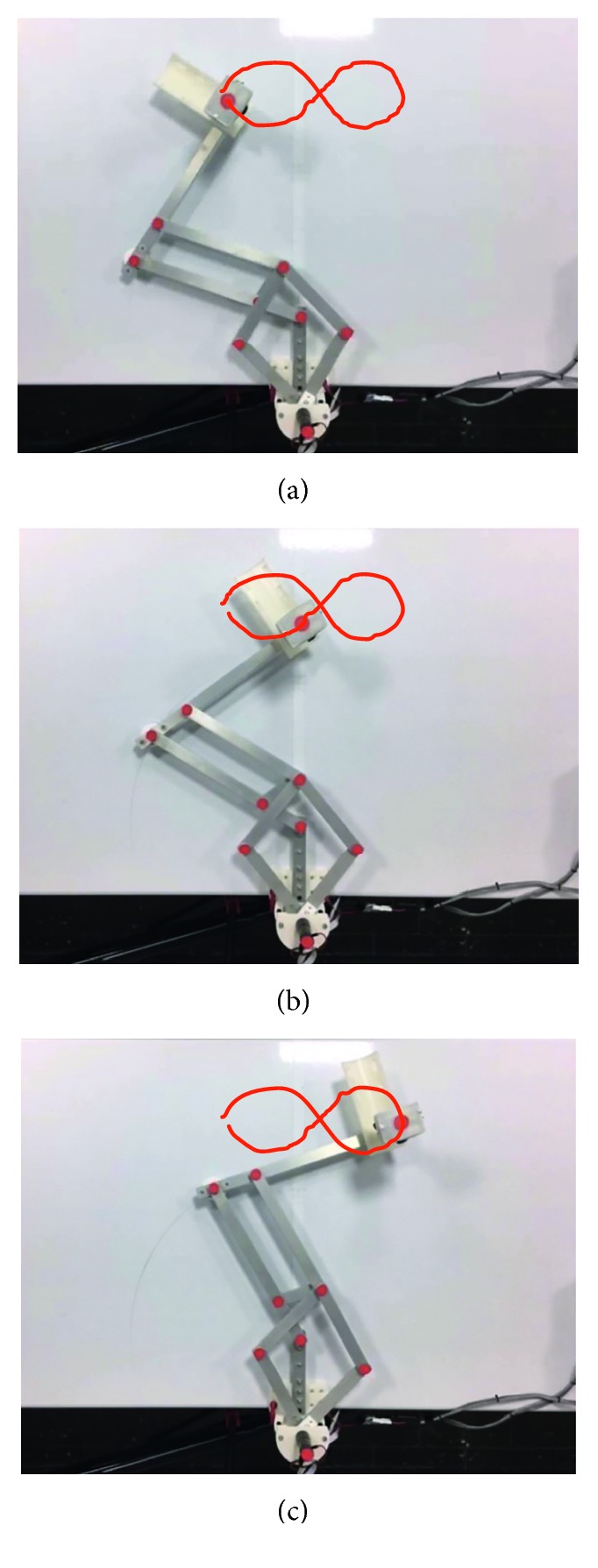
Some snapshots of test no. 2 without load during repetition no. 1 together with the trajectory obtained by image processing (in red): (a) the first sample position; (b) the second sample position; (c) the third sample position.

**Figure 19:**
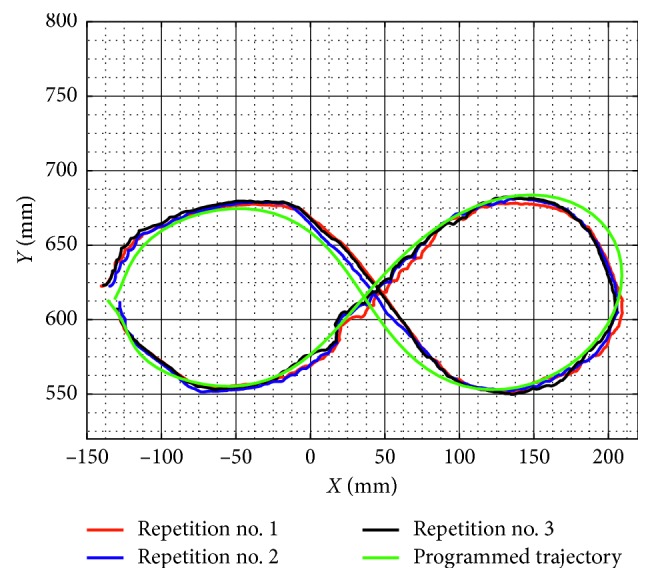
Comparison between the programmed trajectory and the TP trajectories from three repetitions during test no. 2 without load.

**Figure 20:**
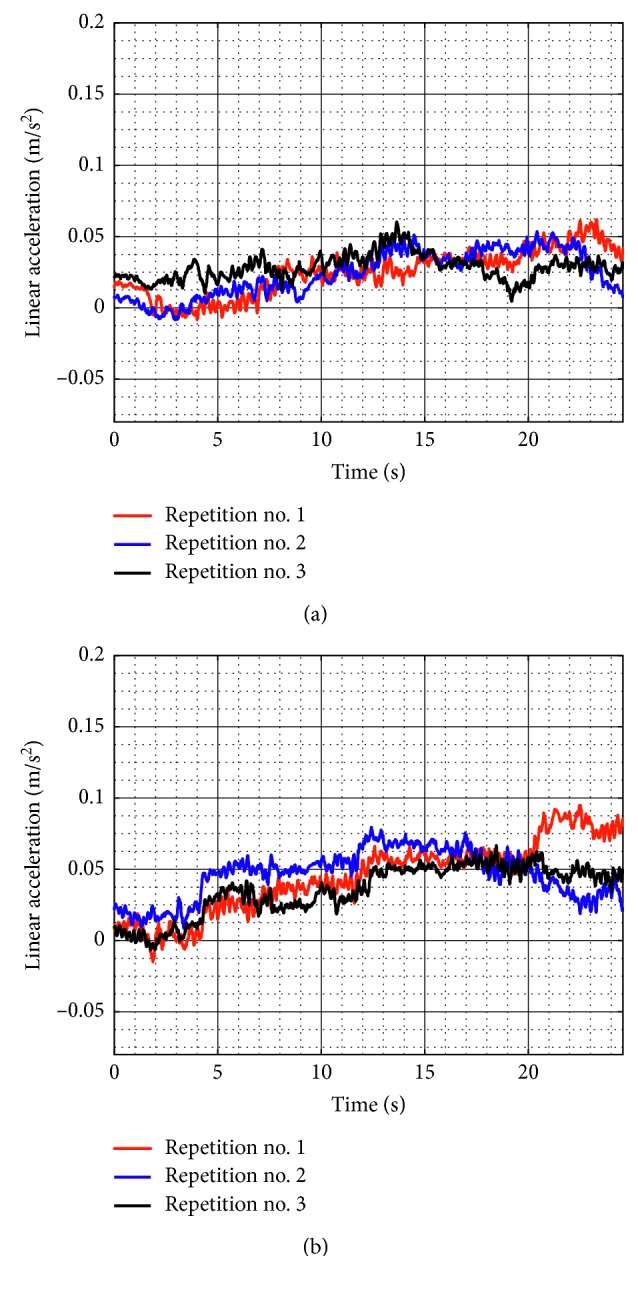
Acquired linear acceleration during test no. 2 without load for the three repetitions seen in Figure 19: (a) *X* linear acceleration; (b) *Y* linear acceleration.

Test no. 2 has been carried out without load during three repetitions. In Figure 18 are shown some snapshots of NURSE when it is performing repetition no. 1 together with the trajectory acquired from the TP.  Figure 19 shows the trajectories acquired from the TP during repetition nos. 1, 2, and 3 and the programmed one. As seen in Figure 19, the trajectories performed by the NURSE to trace the number *8* are close to the programmed one with a maximum deviation of 16 mm. The deviation in test no. 2 is greater than the deviation in test no. 1 since the *8* shape has more changes in direction and is being more complex to perform than the trajectory for horizontal shoulder flexion. On the contrary, the backlash of NURSE wheels can affect the motion more when it has several changes of direction than when it maintains a same direction. However, the repeatability deviation between the trajectories performed by NURSE has a maximum value of 8.22 mm for test no. 2 without load. Despite the fact that the wheels backlash can affect the trajectory shape during test no. 2, the linear accelerations acquired from the TP show that the motion remains smooth as seen in Figure 20, where the linear accelerations in *X* have a maximum value of 0.062 m/s^2^ and a minimum value of −0.008 m/s^2^ and linear accelerations in *Y* have a maximum value of 0.095 m/s^2^ and a minimum value of −0.015 m/s^2^. As seen in Figure 20, the linear acceleration values during test no. 2 have remained in the same range than the linear accelerations during test no. 1.

**Figure 21:**
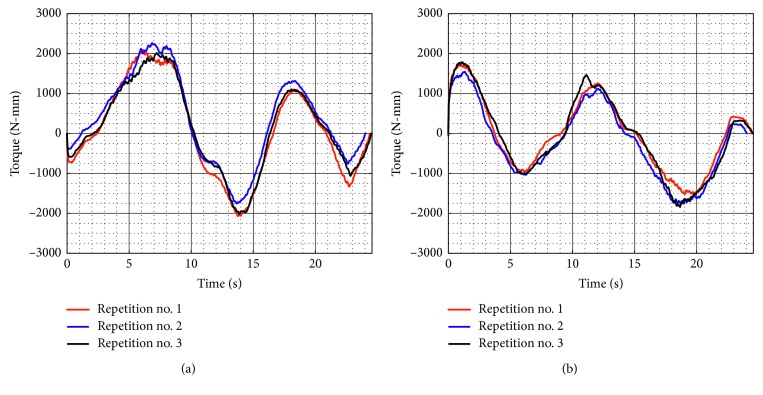
Computed torques during test no. 2 without load for the three repetitions seen in Figure 19: (a) Motor 1; (b) Motor 2.

**Figure 22:**
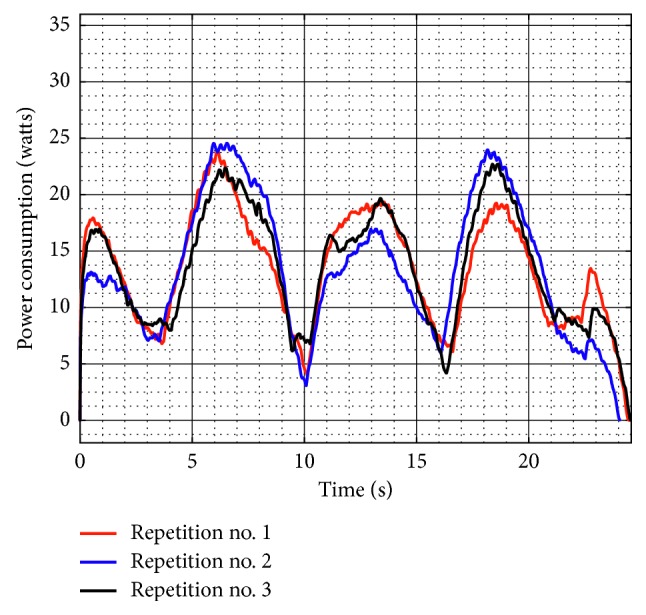
Computed power consumption during test no. 2 without load for the three repetitions seen in Figure 19.

Figure 21 shows the torque required by Motor 1 and Motor 2 during test no. 2 without load. Motor 1 reaches a maximum torque of 2,264 N-mm, and Motor 2 reaches a maximum torque of 1,840 N-mm. As seen in Figure 21, the torques reached by the motors without load during test no. 2 remain in the same range than the torques in test no. 1 without load. Therefore, it shows that when NURSE is unloaded, it requires a similar force to perform the trajectory for horizontal shoulder flexion than it requires to perform the number *8*. The latter is confirmed also by the power consumption that presents a maximum value of 24.510 W (Figure 22). The power consumption during test no. 2 without load increases only 1.380 W with respect to the value in test no. 1 without load. Therefore, NURSE maintains low-power consumption while tracing the number *8*.

**Figure 23:**
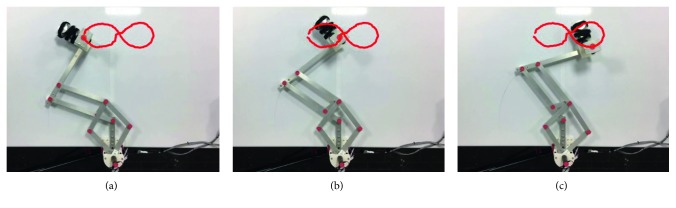
Some snapshots of test no. 2 with a load of 520 g during repetition no. 1 together with the trajectory obtained by image processing (in red): (a) the first sample position; (b) the second sample position; (c) the third sample position.

**Figure 24:**
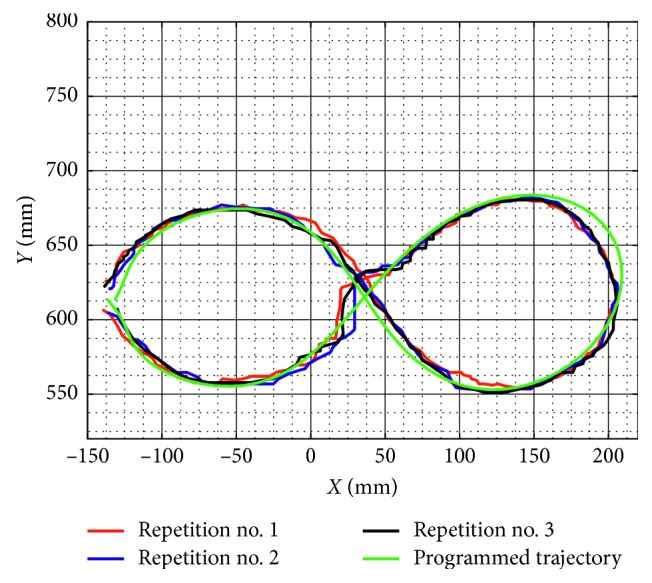
Comparison between the programmed trajectory and the TP trajectories from three repetitions during test no. 2 with a load of 520 g.

**Figure 25:**
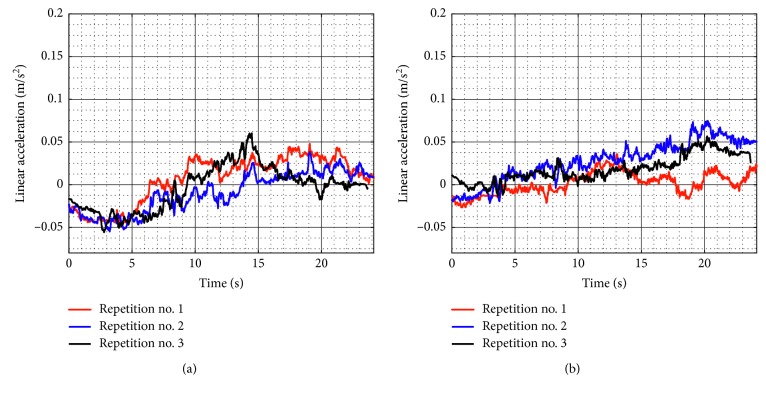
Acquired linear acceleration during test no. 2 with a load of 520 g for the three repetitions seen in Figure 24: (a) *X* linear acceleration; (b) *Y* linear acceleration.

Similarly, test no. 2 has been carried out during three repetitions by using a load of 520 g.  Figure 23 shows some snapshots of NURSE when it is performing repetition no. 1. As seen in Figure 24, the trajectories acquired from the TP during repetition nos. 1, 2, and 3 are close to the programmed one with a maximum deviation of 21 mm. However, the repeatability deviation between the trajectories performed by NURSE has a maximum value of 11.94 mm for test no. 2 with load. Although the wheels backlash introduces deviation in the motion to perform the trajectories, the linear accelerations acquired from the TP show that NURSE motion continues to be smooth with the linear accelerations in *X* having a maximum value of 0.062 m/s^2^ and a minimum value of −0.056 m/s^2^ and linear accelerations in *Y* having a maximum value of 0.075 m/s^2^ and a minimum value of −0.026 m/s^2^ (Figure 25). As seen in Figures 9, 15, 20, and 25, the linear accelerations acquired from the TP are maintained around the same range. Therefore, it can be said that NURSE can reproduce the trajectories with a smooth motion during test nos. 1 and 2 with and without load.

**Figure 26:**
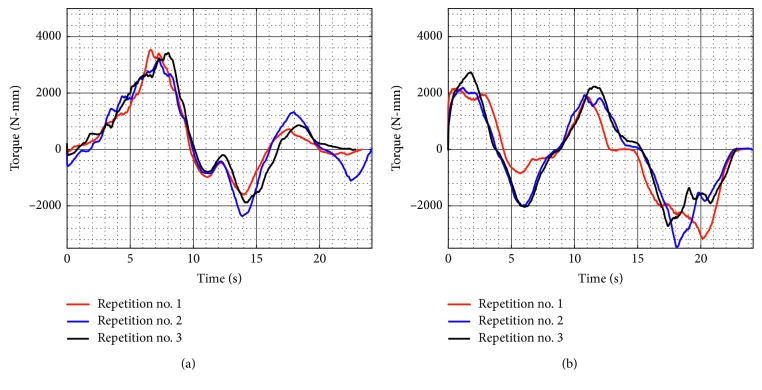
Computed torques during test no. 2 with a load of 520 g for the three repetitions seen in Figure 24: (a) Motor 1; (b) Motor 2.

**Figure 27:**
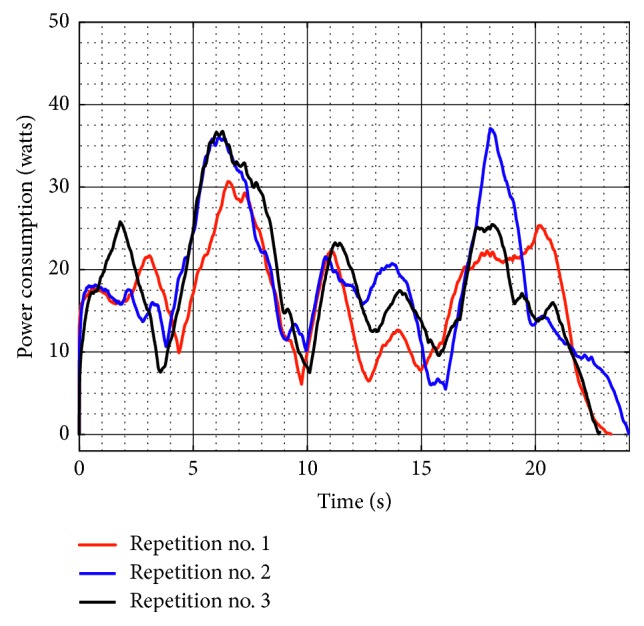
Computed power consumption during test no. 2 with a load of 520 g for the three repetitions seen in Figure 24.

The torque of Motor 1 reached a maximum magnitude of 3,527 N-mm, and the torque of Motor 2 reached a maximum magnitude of 3,464 N-mm, Figure 26. In test no. 2, the torque increases 310 N-mm with respect to the torque when the device is loaded in test no. 1 (Figure 16). It can be said that NURSE needs more force when performing the exercise of test no. 2 than when performing the exercise of test no. 1 both in loaded conditions. However, the torque values are in a range that always can be reached by commercial servomotors. The power consumption has a maximum value of 37.080 W as seen in Figure 27. Therefore, the power consumption increased 6.730 W with respect to the obtained values during test no. 1 with a load as seen in Figure 17. However, NURSE continues to have low-power consumption also to trace the number 8 in loaded conditions.

## 5. Conclusions

NURSE, a device for arm motion assistance, is presented with an experimental characterization. NURSE can assist the motion of both right and left human arms during a rehabilitation therapy or during the arm exercise for elderly people. The NURSE behaviour has been characterized by performing tests of several exercises for upper limb rehabilitation or training, whereas in this paper, two significant ones have been discussed. The tests have successfully been carried out without and with load by looking at trajectory tracking, linear acceleration, torque, and power consumption. The examined trajectories during the tests show that NURSE is able to perform a trajectory near to the programmed one with a minimum deviation of 16 mm when it is unloaded and a maximum deviation of 21 mm in the loaded condition. The trajectories performed by NURSE during reported test no. 1 have a satisfactory maximum repeatability deviation of 3 mm when it is unloaded and 4.5 mm when it is loaded. The trajectories performed by NURSE during reported test no. 2 have a satisfactory maximum repeatability deviation of 8.22 mm between them when it is unloaded and 11.94 mm when it is loaded. The linear accelerations during test nos. 1 and 2 have been successfully measured within a satisfactory range of minimum −0.008 m/s^2^ and maximum 0.095 m/s^2^ with a smooth NURSE motion. The NURSE motors operated with a maximum torque of 3,527 N-mm occurring during test no. 2 with load as a feasible result for commercial servomotors. NURSE worked with low-power consumption without and with a load. The maximum power consumption has been 37.080 W and it has been reached during test no. 2 with load. The experimental results show that NURSE is capable of reproducing successfully different exercises with a smooth motion and a proper low-power consumption.

## Figures and Tables

**Table 1:** Experiment to test NURSE.

Test no.	Description	Inputs	Outputs
1	Perform exercise no. 1	*X*, *Y*	*X*_m_, *Y*_m_, *a*_*x*_, *a*_*y*_, *p*, *τ*_1_, and *τ*_2_
2	Perform exercise no. 2		
